# Supplementary material for: Nickel nanoparticles set a new record of strength
Source: Nat Commun. 2018 Oct 5;9:4102. doi: 10.1038/s41467-018-06575-6 (PMC6173750; doi:10.1038/s41467-018-06575-6)
Supplement: Supplementary file 1 — Supplementary Information [file 41467_2018_6575_MOESM1_ESM.pdf]

Supplementary Information

Nickel nanoparticles set a new record of strength

A. Sharma et al.

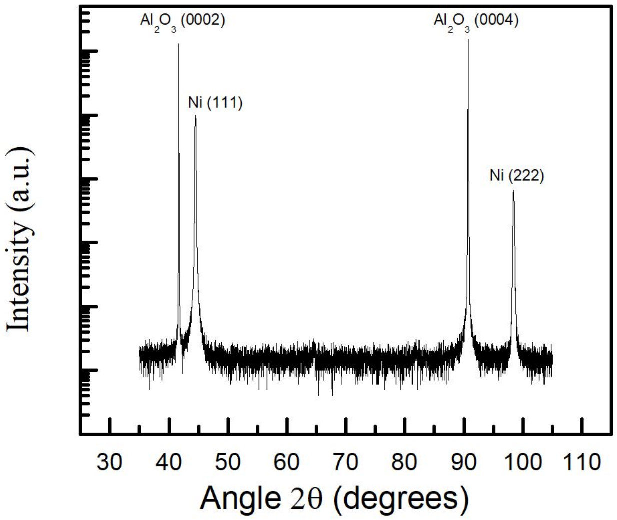

Supplementary Figure 1: XRD scan of a typical Ni nanoparticle on a sapphire substrate after solid state dewetting.

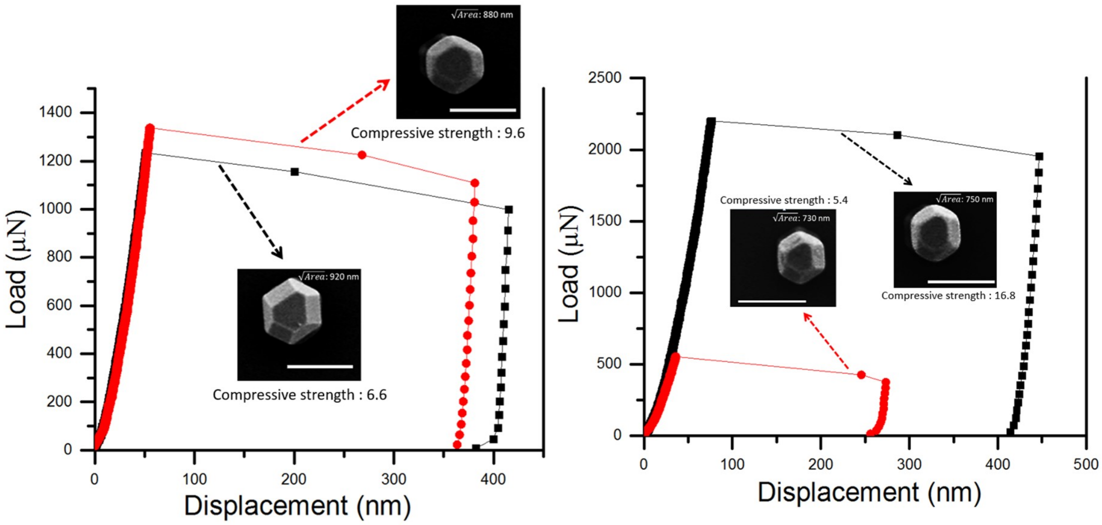

a **b**

Supplementary Figure 2: Experimental demonstration of the particle roundness effect of compressive strength. **a**, **b** Examples of particles with approximately the same size but significantly different strength. The strength correlates with the roundness of the particle shape. **a** larger particles, **b** smaller particles. The compressive strength is indicated in GPa. The scale bars correspond to 1 μm.

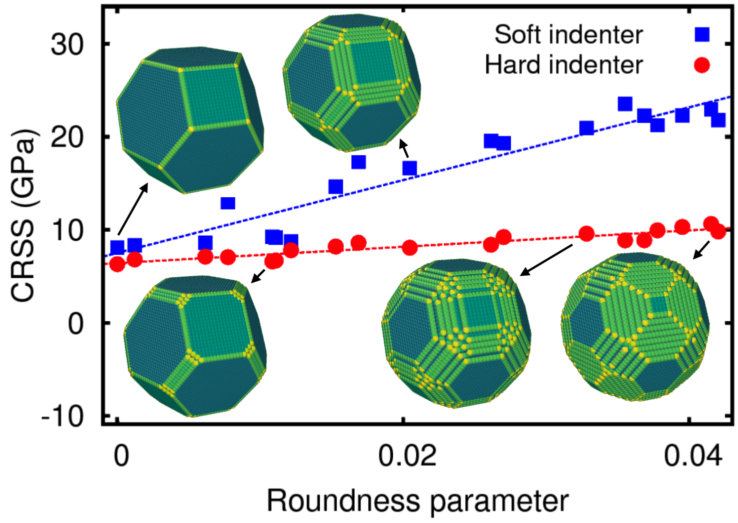

Supplementary Figure 3: Effect of particle roundness on compressive strength in MD simulations. Compressive strength of a 10 nm Ni particle in MD simulations with the hard and soft indenter/substrate as a function of the particle roundness parameter Γ ($\Gamma = 0$ for the ideal Wulff shape). The lines are linear fits that only serve as a guide to the eye. Typical particle shapes are shown for comparison. The atoms are colored according to their energy with brighter colors representing larger energy.

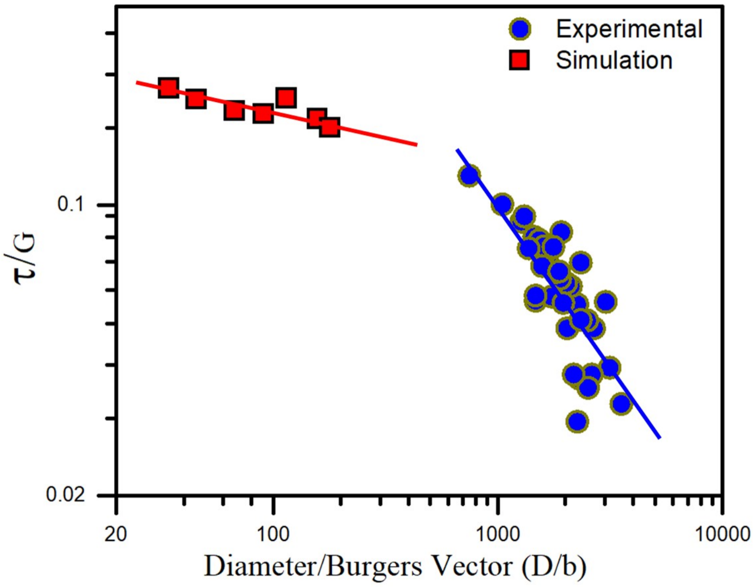

Supplementary Figure 4: Normalized plot of CRSS τ versus diameter D for Ni nanoparticles.The plot compares the experimental results with MD predictions averaged between the hard and soft walls.

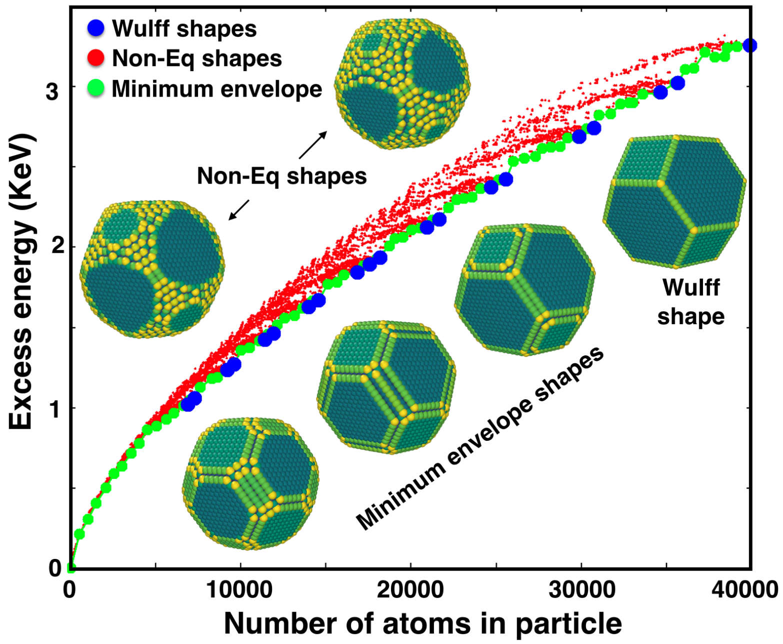

Supplementary Figure 5: Excess energy $\tilde{E}$ of Ni nanoparticles as a function of the number of atoms N. To construct the lower envelope, the data points were binned using $\sim 10^3$ atom windows and selecting the the lowest energy particle within each window. Representative structures of equilibrium (lower envelope) and non-equilibrium particles are shown for comparison. The atoms are colored according to their energy with brighter colors representing larger energy.

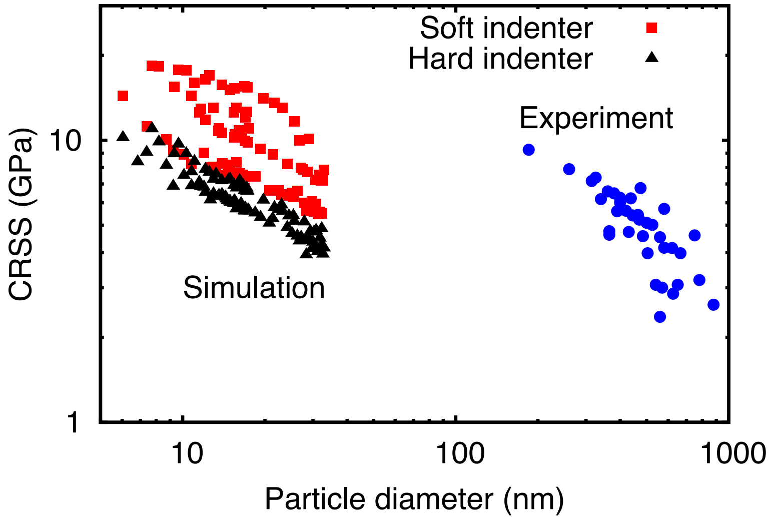

Supplementary Figure 6: Comparison of simulated and experimental compressive strengths of Ni nanoparticles as a function of the particle diameter. The simulations were performed for equilibrium shaped particles corresponding to the lower envelope in Supplementary Fig. 5 using either hard or soft walls for particle compression.

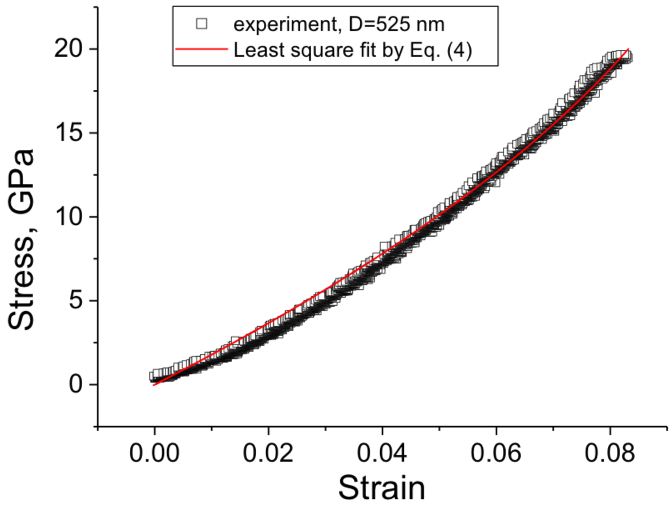

Supplementary Figure 7: Typical elastic part of a stress-strain curve measured on a particle with the projected diameter of $D = 525$ nm (open squares) and the respective least squares fit by Eq. (4) (red line).

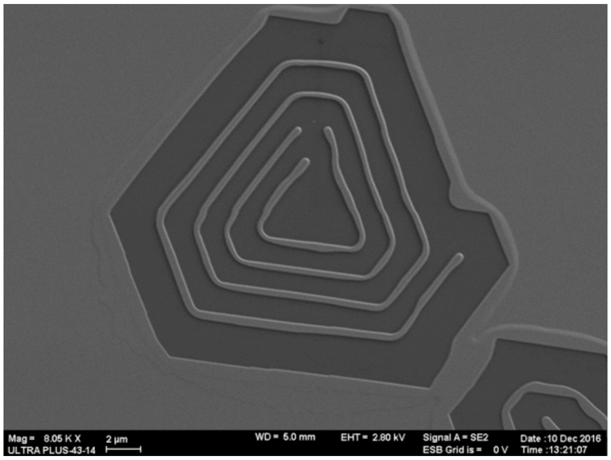

Supplementary Figure 8: Spiral structure formed by solid state dewetting of Ni (22 nm)-Fe (5.3 nm) bilayer deposited on a basal plane-oriented sapphire substrate. The scale bars corresponds to 2 µm.

Supplementary Note 1: Nonlinear elasticity of Ni nanoparticles

The non-linear elastic behavior of the stress-strain curves illustrated in Fig. 3 in the main text was caused by the exceptionally high stresses reached inside the Ni particles during the compression tests. Here we demonstrate that most of the observed non-linear elasticity can be accounted for by taking into account the non-linearity of elastic properties of FCC Ni and the machine compliance of the testing setup. We employ the standard description of non-linear uniaxial deformation [1],

$$\varepsilon = \frac{\sigma}{E_0} - \delta \left(\frac{\sigma}{E_0}\right)^2, \tag{1}$$

where ε, σ, and E_0 are the elastic strain, stress, and the uniaxial Young modulus, respectively. The parameter δ represents the contribution of non-linearity to the strain. For Ni, $\delta \approx 5.3$ [1, 2], and for compression in the [111] direction, $E_0 \approx 259$ GPa [3]. To account for the machine compliance (which is assumed to be linear-elastic because of the low absolute values of loads), another term has to be added to the right hand side of Eq. (1):

$$\varepsilon = \frac{\sigma}{E_0} - \delta \left(\frac{\sigma}{E_0}\right)^2 + \frac{\sigma S}{kh} = \frac{\sigma}{E_{\text{eff}}} - \delta \left(\frac{\sigma}{E_0}\right)^2, \tag{2}$$

where S, h and k are the area of the upper facet of the Ni particle, the particle height, and the spring constant of the nano-indentation instrument, respectively. We introduced an effective elastic modulus of the particle-instrument system:

$$E_{\text{eff}} = \frac{1}{\dfrac{1}{E_0} + \dfrac{S}{kh}}. \tag{3}$$

Generally, the effective modulus can be considered as a phenomenological parameter taking into account both the machine compliance and elastic properties of the substrate. Based on Eq. (2), the elastic part of the stress-strain curve can be described by the equation

$$\sigma = \frac{E_0}{2\delta}\left(R - \sqrt{R^2 - 4\delta\varepsilon}\right) \tag{4}$$

with $R = E_0/E_{\text{eff}} > 1$.

To assess the validity of Eq. (4), it was fitted to a typical stress-strain curve measured in the experiments using R as an adjustable parameter. Supplementary Fig. 7 demonstrates that a good quality of fit can be achieved with $R = 1.486 \pm 0.001$. Slight deviations at low strains can be attributed to a minor misalignment of the punch with respect to the upper facet, and the effect of the surface oxide layer.

Supplementary Note 2: Potential technological applications of Ni nanoparticles

Some of the potential technological applications are mentioned in the last sentence of the paper. This section provides two additional examples.

It is well-known that addition of hard nanoparticles to lubricant oil reduces the friction coefficient and prevents scuffing during tribological tests (a good case in point is the addition of nano-diamond particles to oil [4]). While the mechanism of this improvement is still under debate, two possibilities are: (i) the ball-bearing effect in which the nanoparticles prevent excessive contact plastic deformation of protruding asperities, replacing it by a combination of elastic deformation and rolling, and (ii) the formation of conform deposit film of nanoparticles agglomerates preventing direct contact and excessive rubbing of tribological surfaces. The super-strong Ni nanoparticles synthesized in our work can contribute to friction reduction according to both of the mentioned mechanisms. Their high strength comparable to that of nano-diamonds may result in the ball-bearing effect, while their plastic collapse and formation of pancake-like discs produces favorable conditions for the formation of conform deposit lubricating film. A positive effect of nanoparticulate Ni additions synthesized by wet chemistry methods to lubricant oil has already been reported in the literature [5]. We believe that super-strong, defect-free single crystalline Ni nanoparticles have an even greater potential for friction reduction.

Moreover, complex, high-aspect ratio single crystalline micro-parts can be produced by templated solid state dewetting of the single crystalline heteroepitaxial Ni films (see Ref. 46 in the main text). We anticipate that such complex micro-parts may exhibit the ultimate strength similarly high to that of the nanoparticles studied in our work. Supplementary Fig. 8 presents an example of a spiral structure obtained during solid state dewetting of Ni(Fe) thin film on sapphire (A. Sharma, E. Rabkin, unpublished work). Such structures can be employed as superelastic micro-springs in micro electromechanical systems (MEMS). The key to such applications is the super-high strength of the respective parts, rather than the mechanism of their plastic collapse.

Supplementary References

1. T.E. Wong, G.C. Johnson, On the effect of elastic nonlinearity in metals, Transactions of ASME 110 (1988) 332-337.

2. R. Bechmann, R.F.S. Hearmon, The third order elastic constants, Landolt-BÃ¶rnstein, vol. III/2, Elastic, piezoelectric, piezo-optic constants, and nonlinear dielectric susceptibilities of crystals, Ed. by K.-H. Hellwege and A.M. Hellwege, Springer-Verlag, Berlin, 1969, pp. 102-125.

3. M. Yamamoto, The elastic constants of nickel single crystals, Journal of the Japan Institute of Metals 6 (1942) 331-338.

4. H.-Y. Chu, W.-C. Hsu, J.-F. Lin, The anti-scuffing performance of diamond nano-particles as an oil additive, Wear 268 (2010) 960-967.

5. S. Qiu, Z. Zhou, J. Dong, G. Chen, Preparation of Ni nanoparticles and evaluation of their tribological performance as potential additives in oils, Journal of Tribology 123 (2001) 441-443.
