# Supplementary material for: Nickel nanoparticles set a new record of strength
Source: Nat Commun. 2018 Oct 5;9:4102. doi: 10.1038/s41467-018-06575-6 (PMC6173750; doi:10.1038/s41467-018-06575-6)
Supplement: Supplementary file 3 — Description of Additional Supplementary Files [file 41467_2018_6575_MOESM3_ESM.pdf]

Description of Additional Supplementary Files

File Name: Supplementary Movie 1
Description: In the movie "part-34" we show experimental compression of a nickel nanoparticle by a flat punch. Notice the sudden collapse of the particle in to a “pancake” at a critical value of the strain.

File Name: Supplementary Movie 2
Description: In the movie "100GPa-Wulff" we show the nucleation of a dislocation within a nickel nano-particle occurring at one of the corner vertices. The data was obtained using molecular dynamics simulations carried out on an ideal Wulff nanoparticle under compression. The particle has the diameter of 20 nm. The dislocation nucleates when the compressive stress reaches 22.8 GPa.

File Name: Supplementary Movie 3
Description: Finally, in the movie "100GPa-round" we show homogeneous nucleation of a dislocation inside a rounded nano-particle. The atoms are simulated using molecular dynamics and the particle is under compression. The particle has the diameter of 20 nm and the roundness of 0.024 (see paper). The dislocation nucleates when the compressive stress reaches 70.1 GPa.
